# Social support and pre-operative anxiety in patients undergoing elective surgical procedures: A systematic review and meta-analysis

**DOI:** 10.1177/13591053221116969

**Published:** 2022-09-01

**Authors:** Xiu Ling Florence Kok, J Timothy Newton, Elinor M Jones, Susan J Cunningham

**Affiliations:** 1 UCL Eastman Dental Institute, UK; 2 King’s College London, UK; 3 University College London, UK

**Keywords:** anxiety, social network, social support, surgery, systematic review

## Abstract

Pre-operative anxiety may adversely affect post-operative recovery and treatment satisfaction. This systematic review assessed the impact of social support on pre-operative anxiety in elective surgery patients. MEDLINE via Ovid, Embase, PsycINFO, Web of Science, CINAHL Plus, Emcare and LILACS were searched for publications (1950–2021). Fourteen studies were included for descriptive analysis and five for meta-analysis. The pooled estimate in the meta-analysis was *r* = −0.372 (95% CI: −0.578 to −0.122). Stronger social support was weakly associated with reduced pre-operative anxiety, but the quality of available evidence was low. The findings suggest potential benefit in enhancing utilisation of support networks before elective surgery.

## Introduction

Elective surgery encompasses non-emergency procedures that can be delayed for at least 24 hours (Department of Health Government of Western Australia, accessed 14 May 2019; Royal College of Surgeons of England, accessed 14 May 2019). It is not uncommon for patients awaiting elective surgery to feel anxious, and the prevalence of pre-operative anxiety in surgical patients has been reported to range from 11% to 89% (Abate et al., 2020; Maranets and Kain, 1999; Oteri et al., 2021). Studies have shown that patients who exhibit increased pre-operative anxiety may experience slower recovery and more post-operative symptoms (Bedaso and Ayalew, 2019; Kil et al., 2012; Walburn et al., 2009). It would therefore be useful to identify factors that may reduce patient anxiety, and one possible factor is the availability of social support.

Social support refers to the provision of physical, financial, and/or psychological help by a network of family, friends, or the larger community in times of need. Research has highlighted the therapeutic impact of socially supportive relationships on both psychological and physical health (Ozbay et al., 2007; Reblin and Uchino, 2008; Vaingankar et al., 2020) and social support has also been linked with reduced post-operative pain (Mitchinson et al., 2008) and faster post-operative recovery (Krohne and Slangen, 2005). However, there is a lack of a consensus regarding whether social support has a similar effect on reducing pre-operative anxiety (Bedaso and Ayalew, 2019; Kulik and Mahler, 1989; Okkonen and Vanhanen, 2006). This may be particularly relevant in elective surgery where there might be sufficient time in the pre-operative period for social support effects to be put in place, but this necessitates a more detailed analysis of the available evidence.

This systematic review was therefore conducted to determine the relationship between ‘natural’ social support provided by the patient’s existing social network and pre-operative anxiety in patients undergoing elective surgery.

## Methods

This systematic review was undertaken according to Preferred Reporting Items for Systematic Reviews and Meta-Analyses (PRISMA) guidelines. The protocol for this review was written *a priori* and prospectively registered on the PROSPERO database (CRD42019142722).

### Search strategy

A comprehensive search strategy was developed and seven electronic databases (MEDLINE via Ovid, Embase Classic and Embase, PsycINFO, Web of Science, CINAHL Plus, Emcare and LILACS) were searched for publications from 1950 until December 2021 (for full search strategy, see Supplemental Appendix 1). Reference lists of included studies were hand-searched to identify other relevant grey literature, and authors of included publications were contacted to identify any additional studies or unpublished data that could be incorporated into the review. No language restrictions were applied.

### Study selection

Studies were included in the review if they met the following criteria:

**1. Study design:** Quantitative observational studies or mixed-method studies where it was possible to analyse the quantitative component separately.
**2. Participants:** Patients aged 16 years or older who were consenting to their own treatment and were scheduled for elective surgery under any form of anaesthesia. Exploratory procedures purely for diagnostic purposes were excluded in order to preclude the potentially confounding effects of anxiety due to patients awaiting a diagnosis. The lower age limit of 16 years was selected as this was noted as the youngest age that patients are usually allowed to consent for their own treatment.
**3. Exposure:** Receipt of any form of social support provided by member(s) of the patient’s existing social support network, and assessed by any scale including proxy measures if they were clearly described to reflect social support. Studies that assessed social support provided by clinicians were also included if the support was naturally in existence, as it has been suggested that individuals also consider healthcare professionals as part of their support network (Finfgeld-Connett, 2005; Koivula et al., 2002; Sjolander and Ahlstrom, 2012).
**4. Outcome:** Patient-rated pre-operative anxiety, measured by any scale at any time point prior to scheduled surgery.

All eligibility decisions were performed by two authors (XLFK, SJC) who independently assessed titles and abstracts of all identified studies and evaluated all full texts. Where there was uncertainty or missing data, study authors were contacted for clarification and to obtain the relevant information. Inter-rater reliability for eligibility decisions was evaluated using Cohen’s kappa (κ) statistic, and there was substantial agreement between both authors for all stages. Any disagreements were resolved through discussion between XLFK and SJC, or by the independent advice of a third author (JTN) where necessary.

### Data extraction

Data extraction was conducted independently by both XLFK and SJC using a pre-piloted customised data extraction form. Information recorded included study characteristics, patient demographics, treatment description, measures for social support and pre-operative anxiety, and study results.

### Risk of bias and quality assessment

A modified version of the Newcastle Ottawa Scale (NOS) for cross-sectional studies was utilised to assess the risk of bias for each individual paper (Modesti et al., 2016). The authors of the review made a decision to dichotomise the risk of bias using a cut-off of five stars (out of a maximum score of 10 stars), where studies which scored five stars or less were deemed to be at high risk of bias whilst those scoring more than five were considered to be at low risk. There is, to the best of our knowledge, no clear standardised scoring guidance when utilising the scale and the decision to dichotomise was made taking into consideration the way other studies have used it (Ellis et al., 2017; Luchini et al., 2017; Mata et al., 2015). The Grading of Recommendations Assessment, Development and Evaluation (GRADE) approach was used to determine the strength of the overall body of evidence. Both authors completed these assessments independently, and where there was a difference in rating, the findings were discussed in order to reach a consensus.

### Data synthesis and assessment of heterogeneity

The results of the systematic review were predominantly narrative, involving a structured summary and discussion of the study characteristics and findings. A meta-analysis was also conducted and XLFK and SJC independently assessed studies for inclusion, taking into consideration statistical, clinical and methodological heterogeneity. The criteria used to limit the meta-analysis to certain studies were defined *post-hoc* and were not stated in the original published protocol. Analysis was performed using MedCalc^®^ statistical software, and a test of heterogeneity was also conducted to affirm usage of the random-effects model. Decisions regarding the statistical aspects of the review were undertaken in conjunction with the review statistician (EMJ).

## Results

### Search results

**Figure 1.**
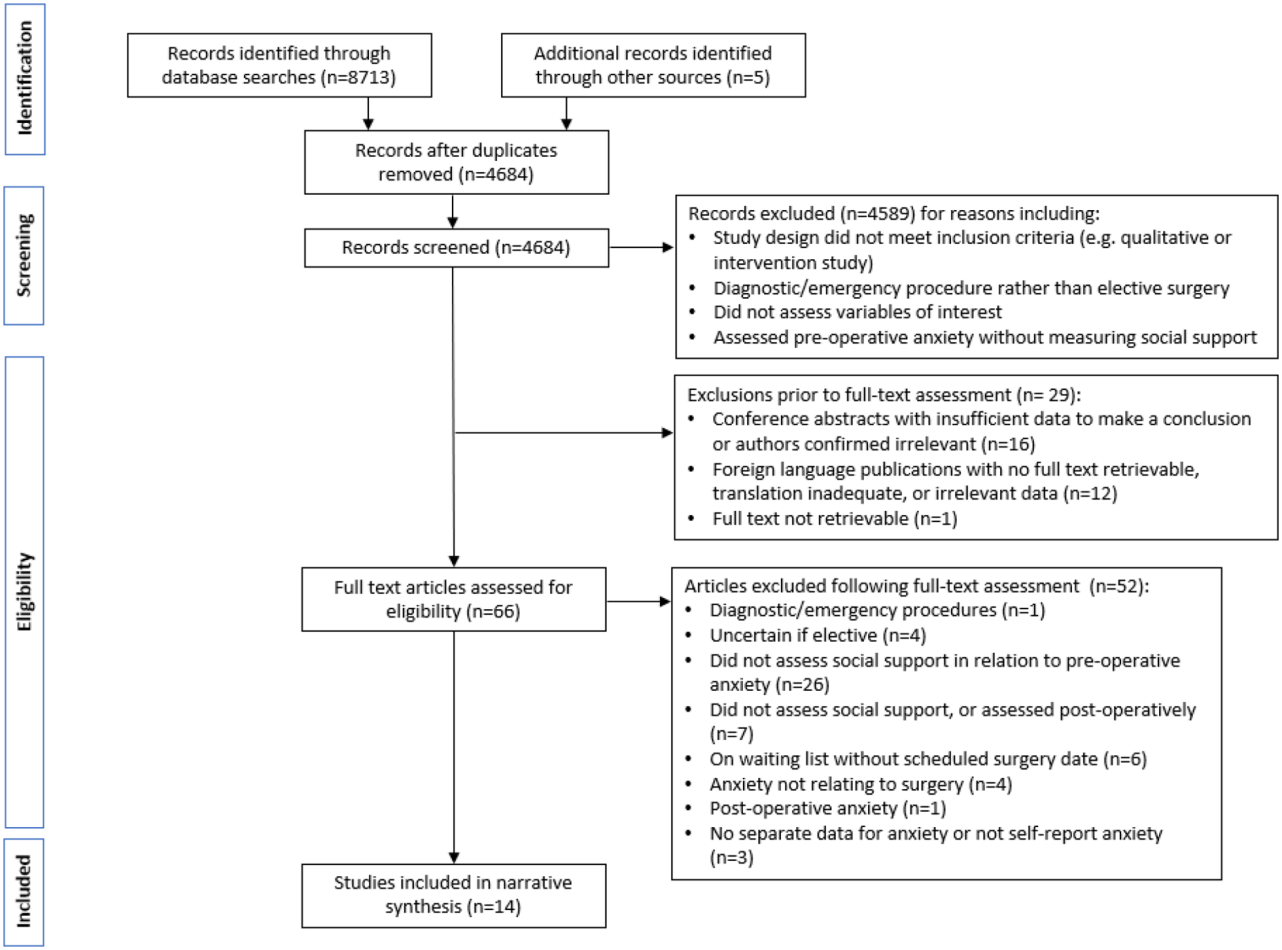
PRISMA flow diagram.

A total of 4684 articles were identified from database searches and through hand searching. Of these, 4670 articles were excluded (Figure 1), and 14 articles were included for final analysis in this systematic review. No foreign language publications were included in the final review as there were considerable difficulties in locating some of these papers or in obtaining accurate translations where it was possible to obtain the full texts.

### Study design and characteristics

All 14 publications were prospective in nature; 11 were cross-sectional and the remaining three were longitudinal in design (Krohne and Slangen, 2005; Nijkamp et al., 2004; Thornton et al., 1997). All studies were, however, considered as cross-sectional for the purpose of this review because only the pre-operative anxiety measurements in the longitudinal studies were relevant to the review question.

Table 1 presents the study characteristics of the 14 studies. Eight publications included patients undergoing different types of elective surgical procedures within the same study, three involved patients scheduled for gynaecological surgery only, and one study each focused on maxillofacial and cataract surgery. The Safdar and Rafiq (2021) paper stated that patients were scheduled for major surgery, but no further details were provided. Two studies specified that surgical procedures were performed under general anaesthesia, a further two under either general or spinal anaesthesia, and one under local anaesthesia. The remaining nine publications did not report on the type of anaesthesia involved.

**Table 1.** Description of included publications.

Author(s)/Year	Country	Population, sample size, study design, funding	Instruments		Type of analysis
			Anxiety	Social support	
Aliche et al. (2020a) ^	Nigeria	• Various elective surgical procedures • *N* = 210 (55.7% female) • Prospective cross-sectional • Funding N/R	• State-Trait Anxiety Inventory, state scale (STAI-S) • 24 hours pre-op	• Multidimensional Scale of Perceived Social Support (MSPSS) • 24 hours pre-op	Pearson’s correlation; Multiple regression
Aliche et al. (2020b) ^	Nigeria	• Various elective surgical procedures • *N* = 210 (55.7% female) • Prospective cross-sectional • Funding N/R	• Anxiety Specific to Surgery Questionnaire (ASSQ)• 24 hours pre-op	• MSPSS • 24 hours pre-op	Pearson’s correlation; Regression from moderation analysis of social support on emotional reactivity and pre-op anxiety
Almalki et al. (2017)	Saudi Arabia	• Elective surgery (GA/spinal) • *N* = 278 (42.1% female) • Prospective cross-sectional • No funding	• Amsterdam Preoperative Anxiety and Information Scale (APAIS) • 24 hours pre-op	• Availability of family support (Yes/No) • 24 hours pre-op	T-test
Bedaso and Ayalew (2019)	Ethiopia	• Various elective surgical procedures • *N* = 402 (43.3% female) • Prospective cross-sectional • No funding	• STAI-S, STAI trait scale (STAI-T) • Specific time point N/R	• Unspecified questionnaire • Specific time point N/R	Bivariate and multivariate logistic regression
Domar et al. (1989)	USA	• Various elective surgical procedures (22% cancer-related) • *N* = 523 (81% female) • Prospective cross-sectional • National Institutes of Health grant funding	• STAI-S, STAI-T • Day of surgery	• Presence of support person on day of surgery, recorded by nurse • Day of surgery	Multiple regression
Gonçalves et al. (2017)	Portugal	• Various elective surgical procedures • *N* = 200 (54.5% female) • Prospective cross-sectional • Funding N/R	• STAI-S (Portuguese) • Inpatient pre-op	• Marital status, expectation of receiving visits in hospital, number of household members • Inpatient pre-op	Mann-Whitney U test; Kruskal-Wallis test; Spearman’s correlation
Ismail et al. (2010)	Hong Kong SAR, China	• Gynaecological surgery (with diagnosed/suspected cancer) • *N* = 170 (100% female) • Prospective cross-sectional • Funding N/R	• STAI-S (Chinese) • Day before surgery	• Medical Outcomes Study Social Support Survey-Chinese (MOS-SSS-C) • Day before surgery	Pearson’s correlation
Krohne and Slangen (2005)	Germany	• Elective maxillofacial surgery (GA) • *N* = 84 (50% female) • Prospective longitudinal* • Funding N/R	• Cognitive, Affective, Somatic Anxiety Scale (CASA) • Averaged (4 measurements) from day before surgery to just before surgery	• Emotional and Informational Support Scales-Operations (EISOP) • After admittance to ward	Pearson’s correlation; Hierarchical regression
Nijkamp et al. (2004)	Netherlands	• Cataract surgery (LA) • *N* = 128 (58% female) • Prospective longitudinal* • Funding N/R	• Short form STAI-S, STAI-T (Dutch), number of items N/R, potentially 6-item version (based on reference quoted) • T1: 1–2 weeks pre-op, T2: just before surgery, only T2 analysed	• Seven-item questionnaire rated on a 4-point scale • 1–2 weeks pre-op	Pearson’s correlation; Multiple linear regression
Peles Bortz et al. (2017)	Israel	• Gynaecological surgery (50% with suspicion of malignancy) • *N* = 100 (100% female) • Prospective cross-sectional • Funding N/R	• STAI-S (Hebrew) • 20–28 hours pre-op	• Tel-Aviv Social Support Questionnaire, 2 additional items on internet support • 20–28 hours pre-op	Pearson’s correlation
Safdar and Rafiq (2021)	Pakistan	• Scheduled for major surgery • *N* = 180 (49% female) • Prospective cross-sectional • Funding N/R	• Depression Anxiety Stress Scales (DASS, Urdu) • Inpatient pre-op	• MSPSS (Urdu) • Inpatient pre-op	Pearson’s correlation
Sharma and Gharti (2019)	Nepal	• Various elective surgical procedures (GA/spinal) • *N* = 442 (74.4% female) • Prospective cross-sectional • University Grant Commission funding	• ASSQ • Day before surgery	• MSPSS • Day before surgery	Pearson’s correlation
Thornton et al. (1997)	UK	• Gynaecological surgery • *N* = 59 (100% female) • Prospective longitudinal* • Funding N/R	• Hospital Anxiety and Depression Scale (HADS) • 3 weeks pre-op	• Family Adaptation and Cohesion Scales (FACES III) • 3 weeks pre-op	Pearson’s correlation
Yilmaz et al. (2012)	Turkey	• Various elective surgical procedures (GA) • *N* = 500 (59.6% female) • Prospective cross-sectional • No funding	• ASSQ • Day before surgery	• MSPSS • Day before surgery	T-test; Multivariable logistic regression

N/R: not reported; GA: general anaesthesia; LA: local anaesthesia.

^ The study authors explained that approximately 75% of the same cohort participated in both studies. This was considered in the interpretation of these studies.

* Only the pre-surgical time point is relevant to this review.

The sample sizes ranged from 59 to 523 patients, and where reported, mean age ranged from 27.52 to 73.2 years. Three papers reported on gynaecological studies and therefore involved only females, whilst the remaining publications generally included more female (54.5%–81%) than male patients. Only one study reported on participant ethnicity, which was Chinese (Ismail et al., 2010), and this may be relevant as different cultural and social norms may affect both social support and anxiety.

### Measurement of pre-operative anxiety and social support

Pre-operative anxiety was generally assessed within 3 weeks prior to surgery. Different anxiety scales were used and it became evident that these fell into two categories: scales that assessed *general anxiety* and those that focused on *surgery-specific anxiety*. The most commonly utilised anxiety scales were the State-Trait Anxiety Inventory [STAI] for general anxiety and the Anxiety Specific to Surgery Questionnaire [ASSQ] for surgery-specific anxiety. Studies that utilised the STAI focused on the relationship between social support and state anxiety. Of the three studies that reported measuring both state and trait anxiety, Domar et al. (1989) and Nijkamp et al. (2004) reported a moderate positive correlation between the two forms of anxiety, but trait anxiety was not explicitly analysed in relation to social support (Bedaso and Ayalew, 2019; Domar et al., 1989; Nijkamp et al., 2004). In studies which evaluated general anxiety, elective surgery patients largely exhibited moderate to high levels of anxiety (Aliche et al., 2020a; Bedaso and Ayalew, 2019; Domar et al., 1989; Gonçalves et al., 2017; Ismail et al., 2010; Thornton et al., 1997) and this was also the case where surgery-specific anxiety was measured (Aliche et al., 2020b; Almalki et al., 2017; Sharma and Gharti, 2019; Yilmaz et al., 2012).

Providers of social support included family, friends, significant others, and medical/nursing staff, but there was generally limited detail provided about the type of social support being measured and the provider of this support. The most commonly used social support scale in the included studies was the Multidimensional Scale of Perceived Social Support [MSPSS]. Three studies employed questionnaires with limited detail of the scales (Almalki et al., 2017; Bedaso and Ayalew, 2019; Nijkamp et al., 2004) and two studies utilised proxy measures for social support: the first assessed the presence of a support person on the day of surgery (Domar et al., 1989), and, in the second, the proxy measure was a combination of marital status, expectation of receiving visits in hospital and number of household members (Gonçalves et al., 2017). Patients generally reported moderate to strong social support (Aliche et al., 2020a, 2020b; Almalki et al., 2017; Bedaso and Ayalew, 2019; Ismail et al., 2010; Krohne and Slangen, 2005; Peles Bortz et al., 2017; Safdar and Rafiq, 2021; Sharma and Gharti, 2019; Yilmaz et al., 2012).

### Effects of social support on pre-operative anxiety (Table 2)

#### Studies which used questionnaires assessing general anxiety

In the seven publications which assessed general anxiety and where social support was directly assessed (i.e. excluding proxy measures), higher social support was associated with lower pre-operative anxiety. This relationship was statistically significant in five of the publications (Aliche et al., 2020a; Bedaso and Ayalew, 2019; Ismail et al., 2010; Peles Bortz et al., 2017; Safdar and Rafiq, 2021). A moderately strong association was noted (*r* = −0.68, *p* < 0.001) where patients from different surgical disciplines were included within the same study (Aliche et al., 2020a), and this retained statistical significance when factors including gender, type of surgery and religious commitment were included in a regression analysis. However, social support was noted to explain only 3% of the variance in pre-operative anxiety. A similar, albeit slightly weaker, correlation (*r* = −0.44, *p* < 0.01) was reported in a Pakistani study involving young adult patients scheduled for major surgery (Safdar and Rafiq, 2021). Where elective surgery patients were classified according to the extent of their social support (Bedaso and Ayalew, 2019), a greater percentage of patients with poor (61.4%) or moderate social support (59.4%) were anxious pre-operatively, as compared with those with strong support (22.5%). After accounting for the potential effects of marital status, education, type of surgical procedure and use of psycho active substances, the adjusted odds ratios also indicated that those with stronger support were less likely to feel anxious (AOR = 0.16, *p* < 0.05) than those with poorer levels of support.

**Table 2.** Results of included publications.

Author (Year)	Association between social support and pre-operative anxiety	Significance	Risk of bias
**Publications that assessed general anxiety**			
Aliche et al. (2020a)	*r* = −0.68 (*p* < 0.001), B* = −0.23 (*p* < 0.01) *Including gender, type of surgery, cognitive reappraisal, expressive suppression, religious commitment	Social support was significantly inversely associated with pre-operative anxiety in both the correlation and regression analysis, but it only explained 3% of the variance in pre-operative anxiety	Low
Bedaso and Ayalew (2019)	Anxious patients by degree of social support: • Poor social support: 35 (61.4%) • Moderate social support: 123 (59.4%) • Strong social support: 31 (22.5%) Strong social support: AOR* = 0.16 (*p* < 0.05) Moderate social support: AOR* = 0.84 (*p* = 0.616) *Including marital status, education, surgical procedure, use of psycho active substances	Patients with strong social support were 84% less likely to be anxious than those with poor social support	High
Domar et al. (1989)	*r* = 0.16 (*p* = 0.006) *Including factors such as age, education, gender, type of procedure	Presence of support person was associated with increased anxiety, but it could have been possible that highly anxious patients are more likely to attend with a support person	Low
Gonçalves et al. (2017)	Marital status: *X*^2^ = 5.137 (*p* = 0.162) Expectation of receiving visits: *X*^2^ = 1.211 (*p* = 0.546) Number of household members: *r_s_* = −0.028 (*p* = 0.691)	There were no statistically significant differences in pre-operative anxiety in relation to the three variables used to measure social support	High
Ismail et al. (2010)	*r* = −0.189 (*p* = 0.014) By social support subscales: • Tangible: *r* = −0.149 (*p* = 0.053) • Emotional–informational: *r* = −0.169 (*p* = 0.027) • Affectionate: *r* = −0.161 (*p* = 0.036) • Positive social interaction: *r* = −0.209 (*p* = 0.006)	Inadequate social support was significantly associated with higher anxiety	Low
Nijkamp et al. (2004)	*r* = −0.265 (*p* < 0.01), β* = −0.075 (NS) *Including factors such as age, gender, education, coping strategies	Social support was negatively associated with pre-operative anxiety but this result was only significant in the correlation analysis and not in the regression analysis	High
Peles Bortz et al. (2017)	Overall: *r* = −0.32 (*p* < 0.001) With suspicion of malignancy: *r* = −0.34 (*p* < 0.01) Without suspicion of malignancy: *r* = −0.27 (*p* < 0.05)	Social support was negatively associated with pre-operative anxiety, and this was significant in the entire sample as well as within both subgroups	Low
Safdar and Rafiq (2021)	*r* = −0.44 (*p* < 0.01) By social support subscales: • Family support: *r* = −0.40 (*p* < 0.01) • Friends support: *r* = −0.32 (*p* < 0.01) • Significant others support: *r* = −0.41 (*p* < 0.01)	Stronger social support was significantly associated with reduced pre-operative anxiety	High
Thornton et al. (1997)	Family cohesiveness: *r* = −0.26 (NS) Family adaptability: *r* = −0.13 (NS)	Social support was inversely related to pre-operative anxiety but this relationship was not statistically significant	High
**Publications that assessed anxiety specific to surgery**			
Aliche et al. (2020b)	Family support: *r* = −0.27 (*p* < 0.01), B* = −0.08 (NS) Friends support: *r* = −0.41 (*p* < 0.01), B* = −0.26 (NS) Significant others support: *r* = −0.31 (*p* < 0.01), B* = −0.02 (NS) *Including emotional reactivity, interaction effect of social support and emotional reactivity	Social support was significantly inversely correlated with pre-operative anxiety in all three subscales but lost significance in the moderation analysis accounting for emotional reactivity	Low
Almalki et al. (2017)	Mean anxiety scores if family support available: 16.6 ± 5.8 Mean anxiety scores if family support not available: 19.4 ± 5.7 *t* = 3.217 (*p* = 0.001)	Patients with family support had significantly lower pre-operative anxiety than those without family support	High
Krohne and Slangen (2005)	**Emotional support:** *r* = 0.26 (*p* < 0.05), β* = 0.18 (NS) • Males: *r* = 0.30 (*p* = 0.05), Females: *r* = −0.15 (NS) • Gender × Emotional support: β* = 2.47 (*p* < 0.05) • ANOVA: ➢ Low support: Females reported significantly higher mean anxiety (31.64 ± 9.75) than males (21.01 ± 4.76), *p* < 0.001 ➢ High support: No gender difference was observed (males: 24.35 ± 7.13, females: 27.48 ± 8.18) **Informational support:** *r* = 0.01 (NS), β* = −0.38 (*p* < 0.01) • Gender × Informational support: β* = −0.71 (NS) *Including age, gender, interaction effect of support variables and gender	Informational support exerted a main effect on pre-operative anxiety, with high support patients reporting significantly lower anxiety levels. The influence of emotional support was moderated by gender, and this was statistically significant. Females with low support exhibited the highest anxiety. In the hierarchical regression, social support explained 6.9% of the variance in pre-operative anxiety whilst the interaction of gender and social support variables explained 5.7%	High
Sharma and Gharti (2019)	*r* = −0.133 (*p* = 0.005)	Higher social support was associated with lower pre-operative anxiety and this was statistically significant	High
Yilmaz et al. (2012)	Low anxiety: Mean MSPSS = 72.66 ± 12.43 High anxiety: Mean MSPSS = 60.50 ± 12.13 *t* = 11.068 (*p* < 0.05), B* = −0.095 (*p* = 0.001) *Including gender, education, marital status, extent of operation, age	Patients without strong social support had significantly higher anxiety than those with strong social support	Low

A significant correlation between social support and anxiety was also noted in two publications that reported on patients undergoing gynaecological surgery, but the strength of this relationship was generally weak (Ismail et al., 2010; Peles Bortz et al., 2017). Chinese women with diagnosed, or suspected, gynaecological malignancy were found to be less anxious where they perceived stronger social support (*r* = −0.189, *p* = 0.014). This was also reflected in three of the four individual social support subscales (emotional-informational, affectionate support and positive social interaction), but did not reach statistical significance for tangible support (Ismail et al., 2010). A similarly weak relationship was noted for Israeli women with (*r* = −0.34, *p* < 0.01) and without suspected gynaecological malignancy (*r* = −0.27, *p* < 0.05), and the difference between the two subgroups was not significant. Higher social support was associated with lower anxiety when support was provided by close and extended family, friends, physicians and nurses, but it was significant only for extended family and friends (Peles Bortz et al., 2017).

In the remaining studies, although a relationship between stronger social support and reduced pre-operative anxiety was apparent, the association was not consistently significant. In a cohort of cataract surgery patients (Nijkamp et al., 2004), a bivariate correlation was statistically significant (*r* = −0.265, *p* < 0.01) but when factors including age, gender, education and coping strategies were accounted for in a subsequent regression analysis, the relationship did not retain significance. The study did not publish actual social support scores thus no further conclusions could be drawn. A weak negative correlation was also noted in the study by Thornton et al. (1997), but again this was not statistically significant.

Both studies that used proxy social support measures reported results that contrasted with those directly assessing social support. The presence of a support person on the day of surgery was significantly associated with greater anxiety (*r* = 0.16, *p* = 0.006) (Domar et al., 1989). No significant differences in anxiety were noted when considering marital status, expectation of visits in the hospital, or number of household members (Gonçalves et al., 2017), but it was not clearly specified whether married patients or those who expected to receive hospital visits experienced more or less anxiety. The authors of this review were unable to obtain further information regarding this.

#### Studies which used questionnaires assessing anxiety specific to surgery

Four of the five publications that assessed anxiety specific to surgery found that social support was inversely related to pre-operative anxiety. Yilmaz et al. (2012) noted that the mean MSPSS score for those with low anxiety was significantly higher than in those with greater levels of anxiety and this relationship retained significance in a logistic regression including gender, education, extent of operation, and age (*B* = −0.095, *p* = 0.001). Almalki et al. (2017) found that patients with available family support also had significantly lower mean anxiety than those without, but the findings from this study should be treated with some caution as it was not specified if the reported Amsterdam Preoperative Anxiety and Information Scale (APAIS) scores referred purely to the anxiety component of the scale or a summed total of all anxiety and information items in the scale. Both Sharma and Gharti (2019) and Aliche et al. (2020b) reported a weak negative correlation between social support and pre-operative anxiety. The results of the latter study were reported according to who provided the support, and support from friends was noted to be more strongly correlated with reduced anxiety (*r* = −0.41), than that from family (*r* = −0.27) or significant others (*r* = −0.31). However, these three relationships were not significant when the analysis accounted for other factors including emotional reactivity (Aliche et al., 2020b).

The findings were less straightforward in the study by Krohne and Slangen (2005). A weak but positive correlation between emotional and informational support and anxiety was noted overall; but inclusion of gender in the analysis revealed that females tended to feel less anxious with greater emotional support although this was not significant (*r* = −0.15, NS). Interestingly, the converse was true for males and this was considered significant (*r* = 0.30, *p* = 0.05). In their regression analysis including gender and age, high informational support was significantly associated with reduced anxiety independent of gender (β = −0.38, *p* < 0.01), but the effects of emotional support were moderated by gender (β = 2.47, *p* < 0.05). Social support explained only 6.9% of the variance in pre-operative anxiety, whilst the interaction of social support with gender contributed an additional 5.7% of explained variance.

#### The relationship between social support and pre-operative anxiety by subgroups

**Gender:** Only one study looked specifically at gender and how it influenced the relationship between social support and anxiety (Krohne and Slangen, 2005). This study found that females were more susceptible to feeling anxious if they had inadequate emotional support, whereas this was less of an issue for males. Gender did not appear to affect the relationship between informational support and pre-operative anxiety, however.

**Type of social support:** Results from the Ismail et al. (2010) study suggested that emotional, informational, affectionate support and positive social interaction played a more significant role with regards to reducing pre-operative anxiety than tangible support (e.g. provision of financial assistance, material goods). Krohne and Slangen (2005) also found that informational and emotional support were significantly associated with pre-operative anxiety, but whilst informational support exerted a main effect (i.e. those with higher support reported less anxiety), the direction of influence for emotional support depended on gender, as described above.

**Provider of social support:** In the Peles Bortz et al. (2017) study, social support from extended family and friends was significantly associated with reduced pre-operative anxiety, but was not significant if the support came from close family members. Similarly, in the Aliche et al. (2020b) study, social support from friends was more strongly correlated with reduced pre-operative anxiety compared with support from significant others and family. These relationships were not significant in subsequent regression analyses, but support from friends was still the most strongly correlated out of the three. However, in the Safdar and Rafiq (2021) study, support from family and significant others were more highly correlated with reduced pre-operative anxiety than support from friends, although all three relationships were statistically significant. Thornton et al. (1997) found that support provided by the patient’s partner was weakly correlated with reduced pre-operative anxiety but this was not statistically significant.

**The effect of a cancer diagnosis:** Although a suspected or confirmed diagnosis of cancer did not appear to significantly affect pre-operative anxiety (Domar et al., 1989; Ismail et al., 2010; Peles Bortz et al., 2017), it was linked with greater social support (Peles Bortz et al., 2017). In the Peles Bortz et al. (2017) study, there was no significant difference in the relationship between social support and pre-operative anxiety in patients having gynaecological surgery, regardless of whether or not there was a cancer diagnosis.

### Risk of bias and quality assessment

Based on the modified NOS, six of the 14 included publications were deemed to be at low risk of bias (Aliche et al., 2020a, 2020b; Domar et al., 1989; Ismail et al., 2010; Peles Bortz et al., 2017; Yilmaz et al., 2012) and eight were at high risk of bias (Almalki et al., 2017; Bedaso and Ayalew, 2019; Gonçalves et al., 2017; Krohne and Slangen, 2005; Nijkamp et al., 2004; Safdar and Rafiq, 2021; Sharma and Gharti, 2019; Thornton et al., 1997). The authors had excellent agreement in scoring with the modified NOS.

Only four studies reported a sample size calculation (Almalki et al., 2017; Bedaso and Ayalew, 2019; Peles Bortz et al., 2017; Sharma and Gharti, 2019) but most articles clearly defined or justified their recruitment strategy (Aliche et al., 2020a, 2020b; Bedaso and Ayalew, 2019; Domar et al., 1989; Gonçalves et al., 2017; Ismail et al., 2010; Peles Bortz et al., 2017). Three publications had relatively low participation rates (i.e. a small proportion of those who were eligible participated) (Aliche et al., 2020a, 2020b; Thornton et al., 1997) and there was no description of the response rate in seven publications (Almalki et al., 2017; Gonçalves et al., 2017; Krohne and Slangen, 2005; Nijkamp et al., 2004; Safdar and Rafiq, 2021; Sharma and Gharti, 2019; Yilmaz et al., 2012). Studies gave limited, or no, information on the characteristics of non-responders to allow for comparison with those who did respond. The use of social support scales with unspecified evidence of validity was also noted in three studies (Almalki et al., 2017; Bedaso and Ayalew, 2019; Nijkamp et al., 2004).

Owing to the observational nature of the included studies, the starting point for the GRADE assessment was already ‘Low’. Both authors agreed that there were sufficient concerns regarding the risk of bias in the included studies to warrant downgrading one level. Therefore, the overall quality of evidence was deemed to be ‘Very low’.

### Meta-analysis

**Figure 2.**
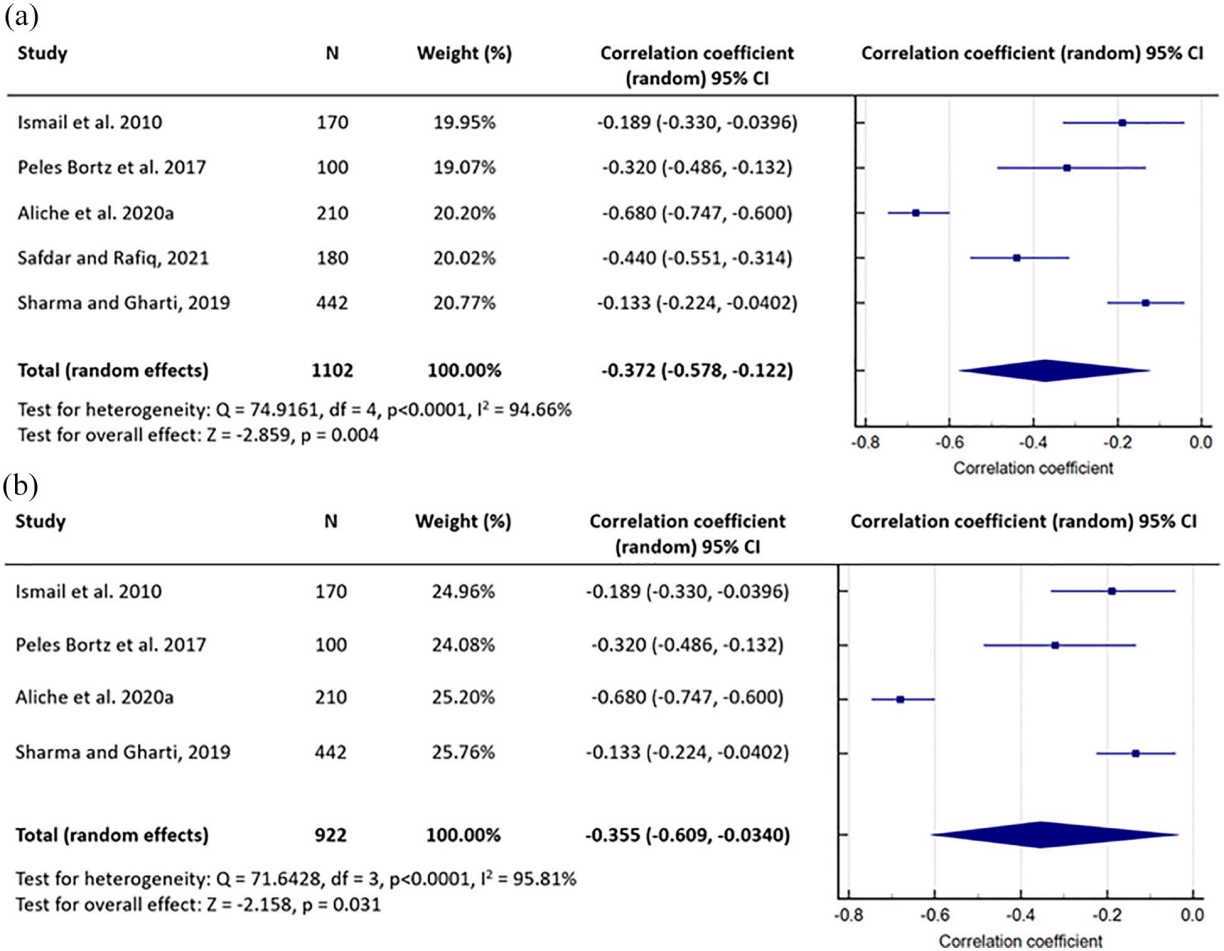
(a) Meta-analysis of the correlation between social support and pre-operative anxiety in elective surgery for all five included studies. (b) Meta-analysis only including those studies which specified that anxiety was measured 1–2 days before surgery.

A meta-analysis was conducted including the results of five studies (Aliche et al., 2020a; Ismail et al., 2010; Peles Bortz et al., 2017; Safdar and Rafiq, 2021; Sharma and Gharti, 2019). The decision to include these studies was made on the basis that social support was directly measured with a valid questionnaire and not a proxy measure, and they reported a Pearson’s correlation coefficient for the entire sample and for total social support scores rather than subscale scores. Four of the five studies measured anxiety within the same time period (approximately 1–2 days before surgery), whilst it was not explicitly stated at what point after hospital admission anxiety was measured in the Safdar and Rafiq (2021) study. The random-effects model was used because of the small number of studies included and the high variability in their estimates. The four studies which assessed general anxiety are presented first in the Forest plot, followed by the study that measured surgery-specific anxiety (Figure 2a). A meta-analysis was also conducted including only the four studies that specified that anxiety was measured 1–2 days before surgery (Figure 2b).

The pooled estimate of the correlation coefficient for social support and pre-operative anxiety was *r* = −0.372 (*p* = 0.004), which suggested that stronger social support was significantly, albeit weakly, associated with reduced anxiety. In four of the five studies, the individual study estimates were relatively similar (Ismail et al., 2010; Peles Bortz et al., 2017; Safdar and Rafiq, 2021; Sharma and Gharti, 2019), but there was a noticeably stronger inverse relationship between social support and anxiety in the Aliche et al. (2020a) study.

## Discussion

Overall, the most commonly used scales were the STAI for anxiety and the MSPSS for social support. Studies that assessed either a general state of anxiety or anxiety specific to the forthcoming surgery were included as this review aimed to explore all concepts of pre-operative anxiety in relation to elective surgery. Whilst there are limitations to including both, it is likely there is some overlap between the two forms of anxiety, in that someone feeling anxious about the surgery may also experience more general anxiety. Interestingly, some of the surgery-specific anxiety questionnaires have been tested against the STAI as a gold standard, and pre-operative ASSQ scores and APAIS anxiety items were found to be highly correlated with STAI state anxiety scores (Karanci and Dirik, 2003; Moerman et al., 1996).

An interesting aspect of this systematic review is that it looked at the relationship between two variables rather than a singular outcome. The results suggested a weak association between stronger social support and reduced pre-operative anxiety, but there was considerable variation in choice of scales used and additional factors included in regression analyses, therefore it was difficult to make further conclusions based on the existing literature. It is possible that the variation in data due to the heterogeneity may have masked those situations where social support is most effective.

It was unclear if social support from any one specific source was more beneficial than another in alleviating pre-operative anxiety, and it is likely, given the geographical diversity in the included studies, that local customs and traditions may impact on the significance of these support networks, contributing to the observed differences. There is some evidence of racial and ethnic differences in preferred sources of social support (Taylor et al., 2013) and it has been proposed that certain cultural norms may influence patients’ willingness to reveal their anxiety (Ayele et al., 2021). Whether there are similar implications when extrapolated to the relationship between social support and pre-operative anxiety is uncertain as only one of the included studies specified the participants’ ethnicity and none investigated its potential effects, but this is an interesting area for future research. Where support from friends appeared to exert a greater effect on reducing anxiety than support from family, it may be that patients viewed family as an expected source of support and might take it for granted, thus the significance of this support may have been less than that from an external source. This would be in agreement with research on psychological wellbeing where support from friends similarly had greater impact than family support, and it was suggested that whilst family support was often more consistent in terms of its longevity, the dynamic nature of this support may lead to periods of reduced empathy and support from friends was therefore important in these instances (Secor et al., 2017).

There is some evidence suggesting that patients undergoing general anaesthesia feel more anxious than those undergoing local or regional anaesthesia, and this may reflect the increased risks associated with general anaesthesia (Almalki et al., 2017; Celik and Edipoglu, 2018; Mitchell, 2012). However to the best of our knowledge, the effect that the type of anaesthesia may have on the level of social support patients receive prior to surgery, or the impact of this social support on pre-operative anxiety is unclear as none of the included studies assessed this and there is limited literature from which to draw conclusions. However, it is possible that in a potentially more anxiety-inducing scenario such as with general anaesthesia, the value of social support might be more appreciated and thus have a greater effect on alleviating anxiety. It would therefore be interesting for future studies to assess the impact of type of anaesthesia on this relationship.

Where proxy measures for social support were used, the results did not support there being a positive effect on pre-operative anxiety, and this may highlight a limitation of using proxy measures. For example, the presence of a support person does not necessarily translate to useful support as perceived by the patient. It could be that the support person’s capacity to provide encouragement and/or assistance might be impaired if they were feeling anxious themselves, or it may be that these patients were highly anxious anyway which could explain why they had a support person with them. In the Gonçalves et al. (2017) study, a high percentage of patients expected to be visited and this might have contributed to a ‘ceiling effect’ and the resulting lack of significance. Furthermore, being married or having more household members may not necessarily translate to positive support if the support network is ineffective or viewed as the norm. This would be consistent with previous research suggesting that access to social support is not associated with marital status (Koivula et al., 2002; Yates, 1995).

There was some variability in sample sizes for the studies included in the meta-analysis, but because the random-effects model was more appropriate in view of the heterogeneity in the estimates of the correlation coefficients, sample size had less influence on the pooled estimate. The meta-analysis and Forest plot highlighted that the Aliche et al. (2020a) study was a statistical outlier, which contributed to a stronger overall relationship between the two variables of interest, but it is not entirely clear why a noticeably stronger effect was observed in this particular study. The study included participants from various surgical disciplines, whereas both Ismail et al. (2010) and Peles Bortz et al. (2017) recruited only those undergoing gynaecological surgery and 52.5% of patients in the Sharma and Gharti (2019) publication were also gynaecology patients. It may be that the type of surgery affects the extent to which social support reduces anxiety, but this cannot be confirmed based on the results of the current review. Additionally, the limitation of including only five studies in a meta-analysis should not be overlooked and the findings should be interpreted with caution. Removal of the Safdar and Rafiq (2021) study from the meta-analysis indicated that the time point when social support and anxiety were measured had minimal impact on the robustness of the findings, although again the small number of studies must be acknowledged. It is possible that patients in the Safdar and Rafiq (2021) study also completed the questionnaires within the same time frame as the other studies in the meta-analysis, but the specific time point was not stated and the review authors were unable to obtain further information on this. Overall, there were more women than men in the meta-analysis, and as gender may affect anxiety (Erkilic et al., 2017; Mulugeta et al., 2018), the results might be more applicable where there is a larger proportion of female patients. Interestingly, in conducting this systematic review, there was surprisingly limited emphasis on the impact of gender on the relationship between the two key variables, and this would be relevant to note for future research.

In reviews of this type which are not studying the effects of an actual intervention, there is often a higher risk of bias, in part because of the observational nature of the research, but this does not necessarily suggest that the evidence is not useful for such a review question where relevant studies would naturally be observational rather than, for example, randomised controlled trials. The lack of information about the sampling strategy in some of the studies and the reported use of convenience sampling in others raises some concern over potential selection bias. This, combined with the voluntary nature of participation, means that there is a possibility that the resultant sample may not be fully representative of the population and may reduce the generalisability of the results. Cultural differences and social situations may affect an individual’s willingness to participate, and it is uncertain if the observed trend in the relationship between social support and pre-operative anxiety would be similar for those who chose not to respond. However, the general lack of information on non-responders, such as their reasons for not participating, precludes further conclusions. Most of the studies that did report on participation and response rates indicated that this was high, however. As data collection in all included studies was conducted prospectively, there was a reduced risk of recall bias, although the use of social support scales with unspecified evidence of validity may mean there is still a possibility of some information bias. Overall, there were some limitations which were consistent across a number of studies and which highlight the potential usefulness for future research to consider the use of standardised questionnaires and clear reporting of a set of core information, in order to allow for improved quality of findings and enable more extensive meta-analyses.

Despite the quality of evidence being deemed ‘Very low’ by GRADE, it is felt that the certainty in the evidence is higher than the rating suggests because it is important to note that GRADE ranks observational studies more poorly than randomised controlled trials at the outset. Observational studies start at a ‘Low’ certainty level, which means that it is more difficult for a review including observational studies to have higher levels of certainty with GRADE.

### Limitations

It is acknowledged that cross-sectional studies would preclude inferences of any causal effects between social support and anxiety, but by virtue of the research question the studies were all cross-sectional and the results can therefore only support the existence of an association. The limitations regarding the use of proxy measures have been discussed in the previous section, and although publications utilising proxy measures were included for a comprehensive review on this topic, they were considered separately from the other studies that directly measured social support. Whilst all efforts were made to include foreign language publications, this was ultimately not possible due to difficulties encountered in obtaining and translating these documents. There may therefore be potentially relevant publications which could not be included in this review.

### Implications for practice and future research

- In future studies, the use of standardised questionnaires for social support and anxiety (e.g. MSPSS and STAI) and the reporting of a core set of information are recommended. For example, this could include a clear description of participant selection and recruitment, the type and provider of social support, and information which may affect both social support and anxiety such as ethnicity, previous surgical experience, and type of anaesthesia. Classification of social support and anxiety scores into low/moderate/high categories would also be helpful for researchers undertaking future systematic reviews where there is sometimes a lack of clear definition regarding what individual scores represent.
- Further exploration regarding the effect of gender on the relationship between social support and pre-operative anxiety in different elective surgical procedures is recommended in future research. How male and female patients effectively utilise this support to alleviate anxiety may differ, and gaining a better understanding of this may help inform future efforts by clinical teams in enhancing levels of support. For example, there may be a need to tailor the approach differently depending on the gender of the patient.
- It may be helpful for clinicians to identify patients with increased pre-operative anxiety and encourage, or facilitate, improved utilisation of their existing social support network. The clinical team could help patients develop a strong support network from an early stage, such as through increasing the involvement of family and friends in key pre-surgery appointments so that these individuals are invested in the process and may be in a better position to support the patient. The involvement of a psychologist/mental health professional in the pre-operative pathway, where feasible, may also be helpful.

## Conclusion

The results of this systematic review and meta-analysis suggest that there is a probable weak relationship between higher social support and lower pre-operative anxiety in elective surgery patients, but the quality of the available evidence is low in part due to the observational nature of studies included in reviews of this type. Nevertheless, the findings suggest there may be value in clinicians encouraging patients to seek support from their social support network ahead of their forthcoming surgery.

## Supplemental Material

sj-docx-2-hpq-10.1177_13591053221116969 – Supplemental material for Social support and pre-operative anxiety in patients undergoing elective surgical procedures: A systematic review and meta-analysis Click here for additional data file. Supplemental material, sj-docx-2-hpq-10.1177_13591053221116969 for Social support and pre-operative anxiety in patients undergoing elective surgical procedures: A systematic review and meta-analysis by Xiu Ling Florence Kok, J Timothy Newton, Elinor M Jones and Susan J Cunningham in Journal of Health Psychology

sj-pdf-1-hpq-10.1177_13591053221116969 – for Social support and pre-operative anxiety in patients undergoing elective surgical procedures: A systematic review and meta-analysis Click here for additional data file. sj-pdf-1-hpq-10.1177_13591053221116969 for Social support and pre-operative anxiety in patients undergoing elective surgical procedures: A systematic review and meta-analysis by Xiu Ling Florence Kok, J Timothy Newton, Elinor M Jones and Susan J Cunningham in Journal of Health Psychology This article is distributed under the terms of the Creative
Commons Attribution 4.0 License ( http://www.creativecommons.org/licenses/by/4.0/) which
permits any use, reproduction and distribution of the work without
further permission provided the original work is attributed as specified
on the SAGE and Open Access pages ( https://us.sagepub.com/en-us/nam/open-access-at-sage).

## References

[bibr1-13591053221116969] Abate SM Chekol YA Basu B (2020) Global prevalence and determinants of preoperative anxiety among surgical patients: A systematic review and meta-analysis. International Journal of Surgery Open 25: 6–16. 10.1016/j.ijso.2020.08.006 PMC7440086 34568611

[bibr2-13591053221116969] Aliche JC Ifeagwazi CM Chukwuorji JC , et al (2020a) Roles of religious commitment, emotion regulation and social support in preoperative anxiety. Journal of Religion and Health 59(2): 905–919. 30145628 10.1007/s10943-018-0693-0

[bibr3-13591053221116969] Aliche JC Ifeagwazi CM Eze JE (2020b) Emotional reactivity and surgical anxiety. The protective nature of perceived social support. Psychology, Health & Medicine 25(4): 434–445. 10.1080/13548506.2019.1668030 31526147

[bibr4-13591053221116969] Almalki MS Hakami OAO Al-Amri AM (2017) Assessment of preoperative anxiety among patients undergoing elective surgery. The Egyptian Journal of Hospital Medicine 69(4): 2329–2333.

[bibr5-13591053221116969] Ayele B Tadesse M Tilahun R , et al (2021) Translation of the Amsterdam Preoperative Anxiety and Information Score (APAIS) into the Amharic version and its validation for evaluation of preoperative anxiety. Ethiopian Journal of Health Sciences 31(2): 349–358. 34158787 10.4314/ejhs.v31i2.18 PMC8188089

[bibr6-13591053221116969] Bedaso A Ayalew M (2019) Preoperative anxiety among adult patients undergoing elective surgery: A prospective survey at a general hospital in Ethiopia. Patient Safety in Surgery 13: 18. 31007718 10.1186/s13037-019-0198-0 PMC6454677

[bibr7-13591053221116969] Celik F Edipoglu IS (2018) Evaluation of preoperative anxiety and fear of anesthesia using APAIS score. European Journal of Medical Research 23(1): 41. 30205837 10.1186/s40001-018-0339-4 PMC6131845

[bibr8-13591053221116969] Department of Health Government of Western Australia. (n.d.). Elective surgery. Available at: https://healthywa.wa.gov.au/Articles/A_E/Elective-surgery (accessed 14 May 2019).

[bibr9-13591053221116969] Domar AD Everett LL Keller MG (1989) Preoperative anxiety: Is it a predictable entity? Anesthesia and Analgesia 69(6): 763–767. 2589657

[bibr10-13591053221116969] Ellis R Blyth R Arnold N , et al (2017) Is there a relationship between impaired median nerve excursion and carpal tunnel syndrome? A systematic review. Journal of Hand Therapy 30: 3–12. 27692791 10.1016/j.jht.2016.09.002

[bibr11-13591053221116969] Erkilic E Kesimci E Soykut C , et al (2017) Factors associated with preoperative anxiety levels of Turkish surgical patients: From a single center in Ankara. Patient Preference and Adherence 11: 291–296. 28280304 10.2147/PPA.S127342 PMC5338979

[bibr12-13591053221116969] Finfgeld-Connett D (2005) Clarification of social support. Journal of Nursing Scholarship 37(1): 4–9. 15813580 10.1111/j.1547-5069.2005.00004.x

[bibr13-13591053221116969] Gonçalves MAR Cerejo MDNR Martins JCA (2017) The influence of the information provided by nurses on preoperative anxiety. Revista de Enfermagem Referência 4(14): 17–26.

[bibr14-13591053221116969] Ismail Z So W Li P (2010) Preoperative uncertainty and anxiety among Chinese patients with gynecologic cancer. Oncology Nursing Forum 37(1): E67–E74. 10.1188/10.ONF.E67-E74 20213960

[bibr15-13591053221116969] Karanci AN Dirik G (2003) Predictors of pre- and postoperative anxiety in emergency surgery patients. Journal of Psychosomatic Research 55(4): 363–369. 14507548 10.1016/s0022-3999(02)00631-1

[bibr16-13591053221116969] Kil HK Kim WO Chung WY , et al (2012) Preoperative anxiety and pain sensitivity are independent predictors of propofol and sevoflurane requirements in general anaesthesia. British Journal of Anaesthesia 108(1): 119–125. 22084330 10.1093/bja/aer305

[bibr17-13591053221116969] Koivula M Paunonen-Ilmonen M Tarkka MT , et al (2002) Social support and its relation to fear and anxiety in patients awaiting coronary artery bypass grafting. Journal of Clinical Nursing 11(5): 622–633. 12201889 10.1046/j.1365-2702.2002.00653.x

[bibr18-13591053221116969] Krohne HW Slangen KE (2005) Influence of social support on adaptation to surgery. Health Psychology 24(1): 101–105. 15631568 10.1037/0278-6133.24.1.101

[bibr19-13591053221116969] Kulik JA Mahler HI (1989) Social support and recovery from surgery. Health Psychology 8(2): 221–238. 2786808 10.1037//0278-6133.8.2.221

[bibr20-13591053221116969] Luchini C Stubbs B Solmi M , et al (2017) Assessing the quality of studies in meta-analyses: Advantages and limitations of the Newcastle Ottawa Scale. World Journal of Meta-Analysis 5(4): 80–84.

[bibr21-13591053221116969] Maranets I Kain ZN (1999) Preoperative anxiety and intraoperative anesthetic requirements. Anesthesia and Analgesia 89(6): 1346–1351. 10589606 10.1097/00000539-199912000-00003

[bibr22-13591053221116969] Mata DA Ramos MA Bansal N , et al (2015) Prevalence of depression and depressive symptoms among resident physicians: A systematic review and meta-analysis. The Journal of the American Medical Association 314(22): 2373–2383. 26647259 10.1001/jama.2015.15845 PMC4866499

[bibr23-13591053221116969] Mitchell M (2012) Influence of gender and anaesthesia type on day surgery anxiety. Journal of Advanced Nursing 68(5): 1014–1025. 21806671 10.1111/j.1365-2648.2011.05801.x

[bibr24-13591053221116969] Mitchinson AR Kim HM Geisser M , et al (2008) Social connectedness and patient recovery after major operations. Journal of the American College of Surgeons 206(2): 292–300. 18222382 10.1016/j.jamcollsurg.2007.08.017

[bibr25-13591053221116969] Modesti PA Reboldi G Cappuccio FP , et al (2016) Panethnic differences in blood pressure in Europe: A systematic review and meta-analysis. PLoS One 11(1): e0147601. 10.1371/journal.pone.0147601 PMC4725677 26808317

[bibr26-13591053221116969] Moerman N van Dam FS Muller MJ , et al (1996) The Amsterdam Preoperative Anxiety and Information Scale (APAIS). Anesthesia and Analgesia 82(3): 445–451. 8623940 10.1097/00000539-199603000-00002

[bibr27-13591053221116969] Mulugeta H Ayana M Sintayehu M , et al (2018) Preoperative anxiety and associated factors among adult surgical patients in Debre Markos and Felege Hiwot referral hospitals, Northwest Ethiopia. BMC Anesthesiology 18(1): 155. 30376809 10.1186/s12871-018-0619-0 PMC6208029

[bibr28-13591053221116969] Nijkamp MD Kenens CA Dijker AJ , et al (2004) Determinants of surgery related anxiety in cataract patients. British Journal of Ophthalmology 88(10): 1310–1314. 15377557 10.1136/bjo.2003.037788 PMC1772346

[bibr29-13591053221116969] Okkonen E Vanhanen H (2006) Family support, living alone, and subjective health of a patient in connection with a coronary artery bypass surgery. Heart & Lung 35(4): 234–244. 16863895 10.1016/j.hrtlng.2005.11.002

[bibr30-13591053221116969] Oteri V Martinelli A Crivellaro E , et al (2021) The impact of preoperative anxiety on patients undergoing brain surgery: A systematic review. Neurosurgical Review 44(6): 3047–3057. 33608828 10.1007/s10143-021-01498-1 PMC8593022

[bibr31-13591053221116969] Ozbay F Johnson DC Dimoulas E , et al (2007) Social support and resilience to stress: From neurobiology to clinical practice. Psychiatry 4(5): 35–40. PMC2921311 20806028

[bibr32-13591053221116969] Peles Bortz A Bluvstein I Bergman L , et al (2017) Anxiety and support resources for Israeli women before gynecological surgery. Women & Health 57(3): 329–341. 26940134 10.1080/03630242.2016.1160964

[bibr33-13591053221116969] Reblin M Uchino BN (2008) Social and emotional support and its implication for health. Current Opinion in Psychiatry 21(2): 201–205. 18332671 10.1097/YCO.0b013e3282f3ad89 PMC2729718

[bibr34-13591053221116969] Royal College of Surgeons of England. (n.d.). Types of surgery. Available at: https://www.rcseng.ac.uk/patient-care/having-surgery/types-of-surgery/ (accessed 14 May 2019).

[bibr35-13591053221116969] Safdar S Rafiq M (2021) Mediating role of perceived social support on mental health problems in pre-operative patients. Anaesthesia Pain & Intensive Care 25(1): 63–70.

[bibr36-13591053221116969] Secor SP Limke-McLean A Wright RW (2017) Whose support matters? Support of friends (but not family) may predict affect and wellbeing of adults faced with negative life events. Journal of Relationships Research 8: e10.

[bibr37-13591053221116969] Sharma S Gharti K (2019) Preoperative anxiety and social support among patients undergoing surgery. Janapriya Journal of Interdisciplinary Studies 8: 149–159.

[bibr38-13591053221116969] Sjolander C Ahlstrom G (2012) The meaning and validation of social support networks for close family of persons with advanced cancer. BMC Nursing 11: 17. 22978508 10.1186/1472-6955-11-17 PMC3488574

[bibr39-13591053221116969] Taylor RJ Chatters LM Woodward AT , et al (2013) Racial and ethnic differences in extended family, friendship, fictive kin and congregational informal support networks. Family Relations 62(4): 609–624. 25089067 10.1111/fare.12030 PMC4116141

[bibr40-13591053221116969] Thornton EW McQueen C Rosser R , et al (1997) A prospective study of changes in negative mood states of women undergoing surgical hysterectomy: The relationship to cognitive predisposition and familial support. Journal of Psychosomatic Obstetrics and Gynaecology 18(1): 22–30. 9138203 10.3109/01674829709085565

[bibr41-13591053221116969] Vaingankar JA Abdin E Chong SA , et al (2020) The association of mental disorders with perceived social support, and the role of marital status: Results from a national cross-sectional survey. Archives of Public Health 78: 108. 33133595 10.1186/s13690-020-00476-1 PMC7592592

[bibr42-13591053221116969] Walburn J Vedhara K Hankins M , et al (2009) Psychological stress and wound healing in humans: A systematic review and meta-analysis. Journal of Psychosomatic Research 67(3): 253–271. 19686881 10.1016/j.jpsychores.2009.04.002

[bibr43-13591053221116969] Yates BC (1995) The relationships among social support and short- and long-term recovery outcomes in men with coronary heart disease. Research in Nursing & Health 18(3): 193–203. 7754090 10.1002/nur.4770180303

[bibr44-13591053221116969] Yilmaz M Sezer H Gürler H , et al (2012) Predictors of preoperative anxiety in surgical inpatients. Journal of Clinical Nursing 21(7-8): 956–964. 21812848 10.1111/j.1365-2702.2011.03799.x

