# Supplementary material for: Social support and pre-operative anxiety in patients undergoing elective surgical procedures: A systematic review and meta-analysis
Source: J Health Psychol. 2022 Sep 1;28(4):309–27. doi: 10.1177/13591053221116969 (PMC10026156; doi:10.1177/13591053221116969)
Supplement: sj-docx-2-hpq-10.1177_13591053221116969 – Supplemental material for Social support and pre-operative anxiety in patients undergoing elective surgical procedures: A systematic review and meta-analysis [file sj-docx-2-hpq-10.1177_13591053221116969.docx]

# APPENDIX 1: SYSTEMATIC REVIEW SEARCH STRATEGY (MEDLINE VIA OVID)

1 Preoperative Care/

2 Preoperative Period/

3 pre-operat*.tw.

4 preoperat*.tw.

5 pre-surg*.tw.

6 presurg*.tw.

7 ((before or prior to or awaiting or undergoing or preparing for or preparation for or candida*) adj5

(surg* or transplant* or graft*)).tw.

8 pre-an$esthetic.tw.

9 prean$esthetic.tw.

10 1 or 2 or 3 or 4 or 5 or 6 or 7 or 8 or 9

11 Anxiety/

12 Panic/

13 panic*.tw.

14 anxi*.tw.

15 worr*.tw.

16 apprehensi*.tw.

17 uneas*.tw.

18 fear*.tw.

19 nervous*.tw.

20 dread.tw.

21 11 or 12 or 13 or 14 or 15 or 16 or 17 or 18 or 19 or 20

22 exp Social Support/

23 Community Networks/

24 community network*.tw.

25 ((social or perceived or emotional or instrumental or appraisal or informational or desired or

enacted or experienced or psychosocial or psychological) adj support).tw.

26 ((famil* or friend* or practition* or an$esthe* or physician* or general practitioner* or peer or

relative* or carer* or caregiver* or spouse* or partner* or significant other or sibling* or nurs*

or hospital* or communit* or clinician* or doctor* or person* or patient*) adj3 (support* or care

or assist* or aid or help)).tw.

27 social ties.tw.

28 Social Networking/

29 social network*.tw.

30 Interpersonal Relations/

31 social relationship*.tw.

32 22 or 23 or 24 or 25 or 26 or 27 or 28 or 29 or 30 or 31

33 10 and 21 and 32

34 limit 33 to yr="1950 -Current"

***************************
