# Supplementary material for: Social support and pre-operative anxiety in patients undergoing elective surgical procedures: A systematic review and meta-analysis
Source: J Health Psychol. 2022 Sep 1;28(4):309–27. doi: 10.1177/13591053221116969 (PMC10026156; doi:10.1177/13591053221116969)
Supplement: sj-pdf-1-hpq-10.1177_13591053221116969 – for Social support and pre-operative anxiety in patients undergoing elective surgical procedures: A systematic review and meta-analysis [file sj-pdf-1-hpq-10.1177_13591053221116969.pdf]

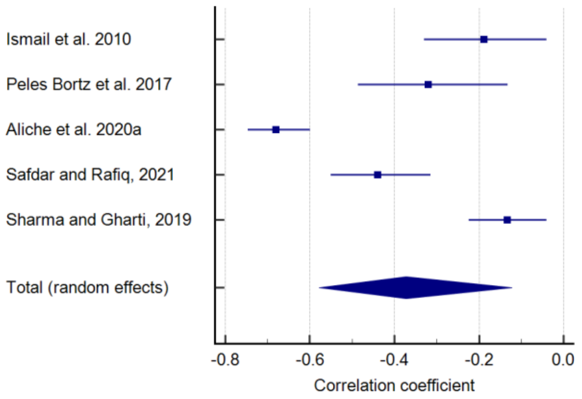

Meta-analysis: correlation

Variable for studies	Study
Variable for number of cases	N
Variable for correlation coefficients	Correlation_coefficient

Study	Sample size	Correlation coefficient	95% CI	z	P	Weight (%)	
						Fixed	Random
Ismail et al. 2010	170	-0.189	-0.330 to -0.0396			15.36	19.95
Peles Bortz et al. 2017	100	-0.320	-0.486 to -0.132			8.92	19.07
Aliche et al. 2020a	210	-0.680	-0.747 to -0.600			19.04	20.20
Safdar and Rafiq, 2021	180	-0.440	-0.551 to -0.314			16.28	20.02
Sharma and Gharti, 2019	442	-0.133	-0.224 to -0.0402			40.39	20.77
Total (fixed effects)	1102	-0.334	-0.386 to -0.281	-11.467	<0.001	100.00	100.00
Total (random effects)	1102	-0.372	-0.578 to -0.122	-2.859	0.004	100.00	100.00

Test for heterogeneity

Q	74.9161
DF	4
Significance level	P < 0.0001
I^2 (inconsistency)	94.66%
95% CI for I^2	90.29 to 97.07

Publication bias

Egger's test	
Intercept	-6.2160
95% CI	-32.4971 to 20.0650
Significance level	P = 0.5063
**Begg's test**	
Kendall's Tau	0.0000
Significance level	P = 1.0000

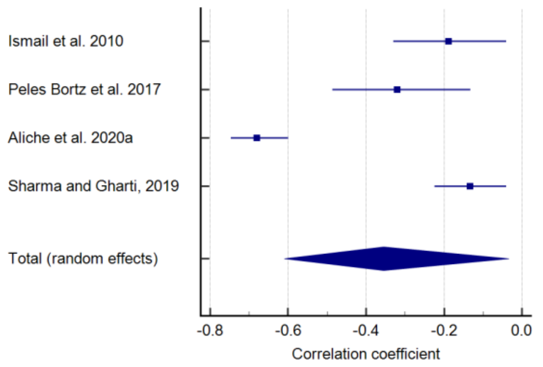

Meta-analysis: correlation

Variable for studies	Study
Variable for number of cases	N
Variable for correlation coefficients	Correlation_coefficient

Study	Sample size	Correlation coefficient	95% CI	z	P	Weight (%)	
						Fixed	Random
Ismail et al. 2010	170	-0.189	-0.330 to -0.0396			18.35	24.96
Peles Bortz et al. 2017	100	-0.320	-0.486 to -0.132			10.66	24.08
Aliche et al. 2020a	210	-0.680	-0.747 to -0.600			22.75	25.20
Sharma and Gharti, 2019	442	-0.133	-0.224 to -0.0402			48.24	25.76
Total (fixed effects)	922	-0.313	-0.370 to -0.253	-9.762	<0.001	100.00	100.00
Total (random effects)	922	-0.355	-0.609 to -0.0340	-2.158	0.031	100.00	100.00

Test for heterogeneity

Q	71.6428
DF	3
Significance level	P < 0.0001
I^2 (inconsistency)	95.81%
95% CI for I^2	92.07 to 97.79

Publication bias

Egger's test	
Intercept	-5.7619
95% CI	-50.3357 to 38.8118
Significance level	P = 0.6340
Begg's test	
Kendall's Tau	-0.3333
Significance level	P = 0.4969
